# Correlation between serum IL-1β and miR-144-3p as well as their prognostic values in LUAD and LUSC patients

**DOI:** 10.18632/oncotarget.13042

**Published:** 2016-11-03

**Authors:** Chen Wu, Bin Xu, You Zhou, Mei Ji, Dachuan Zhang, Jingting Jiang, Changping Wu

**Affiliations:** ^1^ Department of Oncology, The Third Affiliated Hospital of Soochow University, Changzhou, 213003, P.R.China; ^2^ Institute of Cell Therapy, Soochow University, Changzhou, 213003, P.R.China; ^3^ Jiangsu Engineering Research Center for Tumor Immunotherapy, Changzhou, 213003, P.R.China; ^4^ Department of Biological Treatment, The Third Affiliated Hospital of Soochow University, Changzhou, 213003, P.R.China; ^5^ Department of Pathology, The Third Affiliated Hospital of Soochow University, Changzhou, 213003, P.R.China

**Keywords:** LUAD, LUSC, IL-1β, miR-144-3p, prognosis

## Abstract

**Background:**

IL-1β is an essential factor of inflammation initiation, and it also promotes malignant transformation, indicating its tumorigenic property. We aimed to investigate the correlation between IL-1β and miR-144-3p as well as their prognostic values in LUAD and LUSC patients.

**Results:**

The IL-1β level in both LUAD and LUSC patients was significantly higher than that of healthy donors (*P* < 0.001). In both populations, patients with low IL-1β level had better prognosis than high IL-1β level (*P* < 0.001 and *P* = 0.010, respectively). In A549 cells, miR-144 showed the biggest expression change (−4.38 fold) after IL-1β exposure. In LUAD patients, a negative correlation was detected between IL-1β and miR-144-3p (r = –0.540, *P* < 0.001) and the high miR-144-3p group had better prognosis (*P* = 0.003), which was validated by TCGA data. Clinical stage, IL-1β and miR-144-3p were independent risk factors in LUAD patients. *In vitro*, IL-1β and miR-144-3p antagomir could enhance proliferation and miR-144-3p mimics would attenuate the promoting effect of IL-1β.

**Materials and Methods:**

ELISA and qRT-PCR were applied respectively to detected cytokines and miR-144-3p in 129 LUAD, 54 LUSC and 40 healthy donors. Moreover, miRNA array was carried out for miRNA profiling. TCGA database was employed for validation, and follow up data were collected for prognosis evaluation. MTT assay and western-blot were carried out for proliferation evaluation.

**Conclusions:**

In LUAD patients, the serum IL-1β level was correlated with miR-144-3p may affect miR-144-3p at transcriptional level. Both of them were independent risk factors for LUAD prognosis. In addition, IL-1β and miR-144-3p might mediate inflammation-promoted tumorigenesis in LUAD patients.

## INTRODUCTION

Lung cancer remains the leading cause of cancer-related death worldwide. It accounts for approximately 1.58 million deaths in 2015 in USA, and such deaths are projected to continue rising [1]. According to histological characteristics, lung cancer can be categorized into non-small-cell lung cancer (NSCLC) and small-cell lung cancer (SCLC). In NSCLC, lung adenocarcinoma (LUAD) and lung squamous cell carcinoma (LUSC) account for 60% and 25% of this population, respectively [2].

Besides histological differences, many aspects, including genetics, pathogenesis, biological behavior, treatment and prognosis, significantly vary between LUAD and LUSC [3–7]. As a result, it is highly recommended by both scientists and clinical doctors to distinguish them as two individual diseases.

It has been well established that chronic inflammation and infection are pivotal factors of carcinogenesis [8]. Interleukin-1 beta (IL-1β), which is produced by activated macrophages as a proprotein and processed to its active form by Caspase 1, is a member of the IL-1 cytokine family [9]. IL-1β is an important mediator of the inflammatory response and involved in a variety of cell behaviors. The induction of cyclooxygenase-2 (PTGS2/COX2) by IL-1β in the central nervous system (CNS) is found to contribute to inflammatory pain hypersensitivity [10]. Besides, IL-1β promotes malignant transformation and tumor aggressiveness in oral cancer [11], indicating its tumorigenic role.

Recently, mounting evidence has shown that microRNAs (miRNAs) are a novel class of inflammation-associated molecules and involved in multi-stages of tumor development, including initiation, tumor progression, invasion and metastasis [12–14]. Tumor-related miRNAs can be regulated/influenced by various inflammatory stimuli, including cytokines [15–17]. Lin et al. demonstrated that IL-1β upregulates Lin28B by downregulating miR-101 and promotes proliferation and migration of NSCLC cells, defining an IL-1β/miR-101/Lin28B pathway in NSCLC [18].

As NSCLC is a tissue specific tumor, its corresponding treatment strategies are dissimilar widely. Oncologists and scientists currently consider that LUAD and LUSC are two individual diseases with completely different genetic background [3]. In this study, we detected cytokines in both LUAD and LUSC patients and found that the IL-1β levels in both LUAD and LUSC patients were significantly higher than that of healthy donors. Moreover, in both LUAD and LUSC, low IL-1β cases had better prognosis that high IL-1β ones. In addition, miR-144-3p was a potential downstream miRNA correlated with and influenced by IL-1β in LUAD. LUAD patients with high miR-144-3p level had better prognosis. Based on TCGA database, the miR-144-3p level in tumor tissue was crucially higher than that in adjacent area in both LUAD and LUSC. However, miR-144-3p was associated with prognosis only in LUAD population, which was consistent with our results. IL-1β and miR-144-3p suppression could enhance proliferation of LUAD cells. Therefore, our findings suggested a potential link between IL-1β and miR-144-3p, which might be associated with LUAD prognosis, providing new insights into inflammation-promoted tumorigenesis.

## RESULTS

### Comparison of various cytokines in serum from LUAD, LUSC and healthy donors

**Figure 1:**
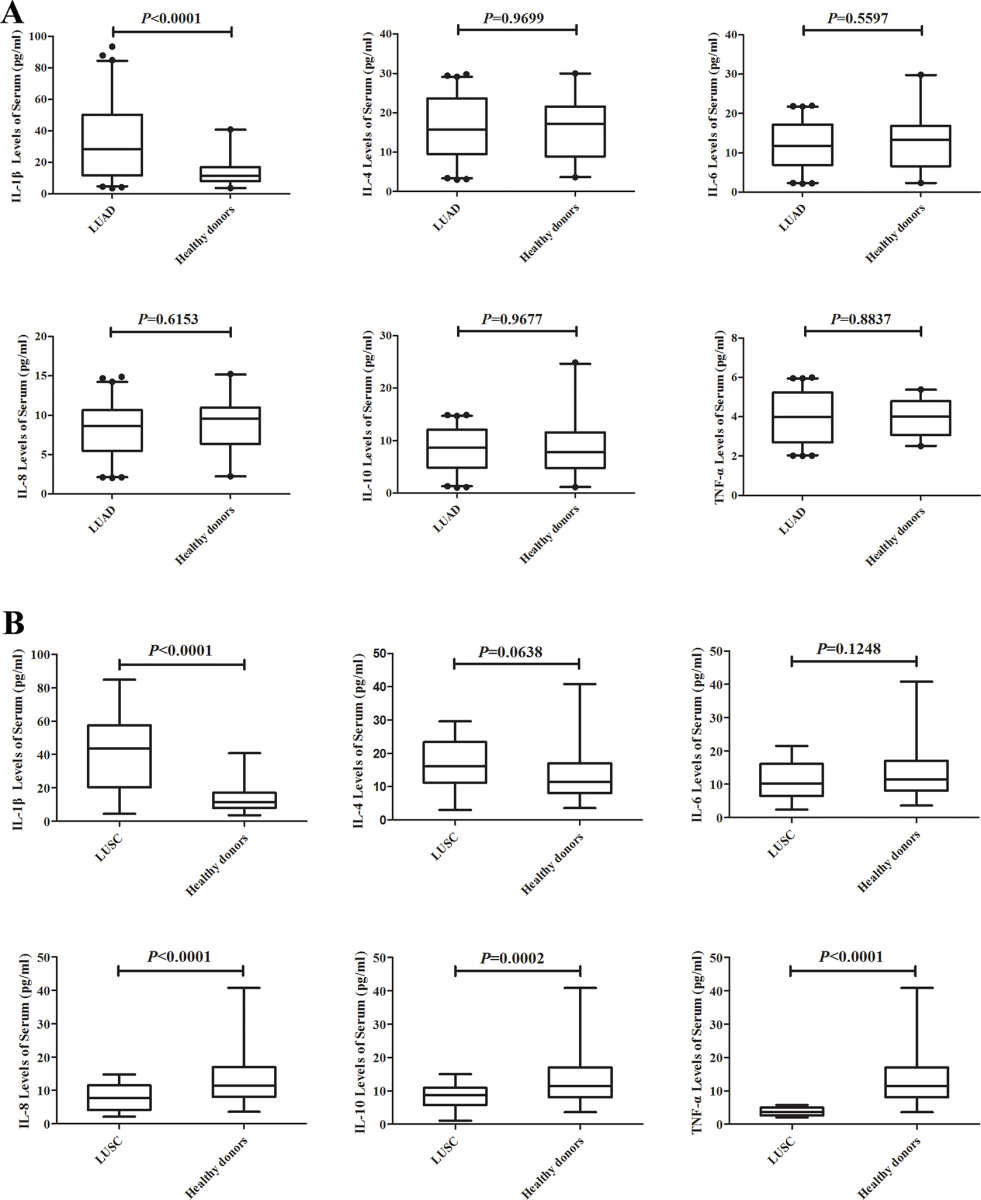
Comparison of various cytokines in serum from a set of 129 LUAD, 54 LUSC and 40 healthy donors IL-1β level in LUAD patients was significantly higher than Healthy donors (*P* < 0.001), whereas IL-4, IL-6, IL-8, IL-10 or TNF-α levels varied slightly between LUAD and healthy donors (**A**); IL-1β level in LUSC patients were significantly higher than healthy donors, while IL-8, IL-10 and TNF-α were lower in LUSC than healthy donors (*P* < 0.001), however, IL-4 and IL-6 levels varied slightly between the two groups (**B**).

A total of six cytokines were detected using ELISA method in LUAD, LUSC patients and healthy donors. The results showed that the IL-1β level in LUAD patients was significantly higher than thatof healthy donors (*P* < 0.001), whereas levels of IL-4, IL-6, IL-8, IL-10 or TNF-α slightly varied between LUAD patients and healthy donors (Figure 1A). The IL-1β level in LUSC patients was significantly higher than that of healthy donors, while levels of IL-8, IL-10 and TNF-α were lower in LUSC than healthy donors (*P* < 0.001). However, IL-4 and IL-6 levels slightly varied between the two groups (Figure 1B).

### Association between IL-1β and clinical features in LUAD and LUSC patients

The IL-1β level was defined as “high” and “low” using median (28.3992 pg/mL and 43.5495 pg/mL in LUAD and LUSC, respectively) as threshold. IL-1β level of LUAD and LUSC patients was analyzed according to age, gender, smoking status and pathological stage, as well as target therapy status in LUAD patients. The results showed that in LUSC patients, the IL-1β level was associated with pathological stage (*P* < 0.05). However, no significant associations were observed between IL-1β level and other above-mentioned clinical features in either LUAD or LUSC population (Table 1).

**Table 1:** Association between IL-1β and clinical features in LUAD and LUSC patients

Characteristics	LUAD				LUSC			
	All patients	IL-1β Low (< median^a^)	IL-1β High (≥ median^a^)	*p*	All patients	IL-1β Low (< median^b^)	IL-1β High (≥ median^b^)	*p*
*n*	129	64 (49.60%)	65 (50.40%)		54	27 (50.00%)	27 (50.00%)	
Age (years)				0.842				0.695
Median (range)	65 (48–85)	65 (49–84)	65 (48–85)		64 (50–85)	64 (50–83)	64 (51–85)	
Gender				0.401				0.761
Male	93 (72.09%)	44 (34.11%)	49 (37.98%)		39 (%72.22)	20 (37.05%)	19 (35.17%)	
Female	36 (27.91%)	20 (15.50%)	16 (12.40%)		15 (27.78%)	7 (12.97%)	8 (14.81%)	
Smoke				0.334				0.248
Yes	61 (47.29%)	33 (25.58%)	28 (21.71%)		36 (66.67%)	20 (37.04%)	16 (29.63%)	
No	68 (52.71%)	31 (24.03%)	37 (28.68%)		18 (33.33%)	7 (12.96%)	11 (20.37%)	
Pathological stage^c^				0.330				0.023
IA	17 (13.18%)	11 (8.53%)	6 (4.65%)		11 (20.37%)	8 (14.81%)	3 (5.56%)	
IB	34 (26.36%)	20 (15.50%)	14 (10.85%)		10 (18.52%)	8 (14.81%)	2 (3.71%)	
IIA	23 (17.83%)	12 (9.30%)	11 (8.53%)		11 (20.37%)	5 (9.26%)	6 (11.11%)	
IIB	32 (24.81%)	13 (10.08%)	19 (14.73%)		13 (24.07%)	5 (9.26%)	8 (14.81%)	
IIIA	11 (8.53%)	4 (3.10%)	7 (5.43%)		8 (14.81%)	1 (1.85%)	7 (12.96%)	
IIIB	12 (9.30%)	4 (3.10%)	8 (6.20%)		1 (1.85%)	0 (0.00%)	1 (1.85%)	
Target therapy				0.537				NT^d^
Yes	65 (50.39%)	34 (26.36%)	31 (24.03%)		NT^d^	NT^d^	NT^d^	
No	64 (49.61%)	30 (23.26%)	34 (26.36%)		NT^d^	NT^d^	NT^d^	

Median^a^ = 28.3992 pg/ml Median^b^ = 43.5495 pg/ml NT^d^ = Not Tested

Pathological stageC was based on NCCN Guidelines Version 3.2011 NSCLC.

### Prognosis evaluation between high and low cytokine levels in LUAD and LUSC patients

**Figure 2:**
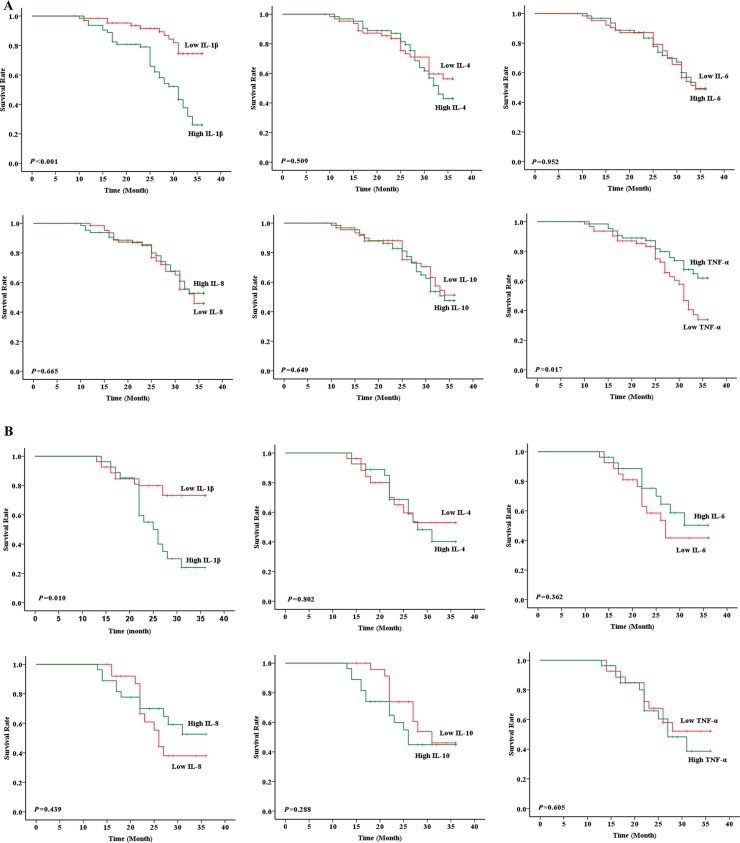
Prognosis evaluation between high and low cytokine levels in LUAD and LUSC patients In LUAD, patients with low IL-1β and high TNF-α levels showed better prognosis than those with high IL-1β (*P* < 0.001) and low TNF-α (*P* = 0.017) levels, respectively, whereas no significant differences in prognosis were observed between patients with high and low IL-4 (*P* = 0.509), IL-6 (*P* = 0.952), IL-8 (*P* = 0.665) or IL-10 (*P* = 0.649) levels (**A**); In LUSC, patients with low IL-1β level had better prognosis than high IL-1β (*P* = 0.010) level. However, no significant prognostic differences were detected between patients with high and low IL-4 (*P* = 0.802), IL-6 (*P* = 0.362), IL-8 (*P* = 0.439), IL-10 (*P* = 0.288) or TNF-α (*P* = 0.605) levels (**B**).

Levels of cytokines were defined as “high” and “low” using medians as thresholds. In both LUAD (Figure 2A) and LUSC (Figure 2B) populations, patients with low IL-1β level showed better prognosis than those with high IL-1β level (*P* < 0.001 and *P* = 0.010, respectively). In LUAD population, patients with high TNF-α level exhibited better prognosis than those with low TNF-α level (*P* = 0.017). No significant differences in prognosis were observed between patients with high and low IL-4, IL-6, IL-8 or IL-10 levels (Figure 2).

### The prognostic value of miR-144-3p and its correlation with IL-1β

**Figure 3:**
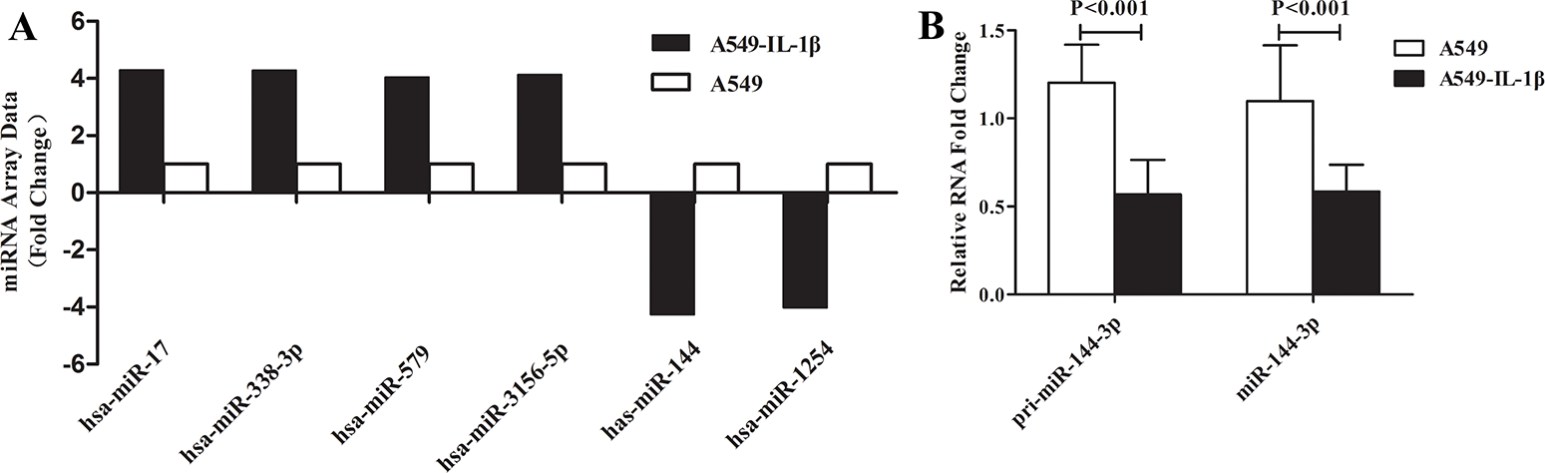
IL-1β was involved in regulation of miR-144-3p at the transcriptional level Via miRNA array, a total of six miRNAs varied greater than 4 folds (including miR-144-3p) in A549 cells after high IL-1β exposure. (**A**); Real-time PCR validated the result of miRNA array and showed that both pri-miR-144-3p and miR-144-3p were down-regulated in A549-IL-1β group (*P* < 0.05, **B**).

Figure 3A reveals that hsa-miR-17, hsa-miR-338-3p, hsa-miR-579, hsa-miR-3156-5p, hsa-miR-144 and hsa-miR-1254 ranked as the top six mostly altered miRNAs after IL-1β exposure in A549 cells, with an expression change greater than ± 4 fold-change, among which hsa-miR-144 showed the biggest change (−4.38 fold). Real-time PCR was carried out to validate the array data, and results showed that both pri-miR-144-3p and miR-144-3p were down-regulated in A549-IL-1β group (*P* < 0.05), indicating that IL-1β could regulate the transcription of miR-144-3p.

**Figure 4:**
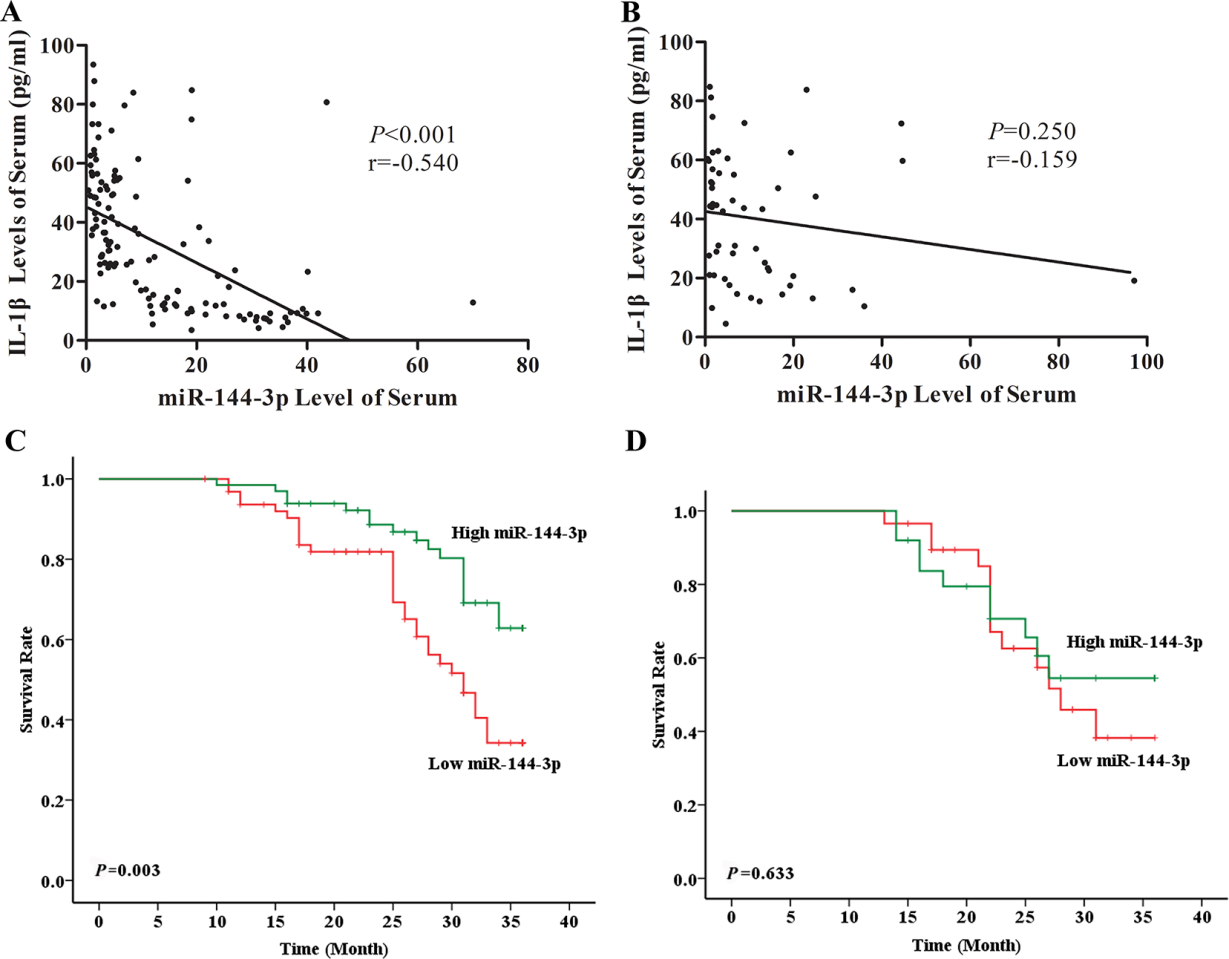
Serum miR-144-3p level was correlated with IL-1β level and associated with prognosis in LUAD Via Pearson correlation analysis, serum level of miR-144-3p was negatively correlated with IL-1β (r = −0.540, *P* < 0.001) (**A**) and LUAD patient with high miR-144-3p level showed significantly better prognosis than the ones with low miR-144-3p level (*P* = 0.003) (**C**); In the contrary, in LUSC patients, serum miR-144-3p level was neither correlated with IL-1β (r = −0.159, *P* = 0.250) (**B**) nor associated with prognosis (*P* = 0.633) (**D**).

**Figure 5:**
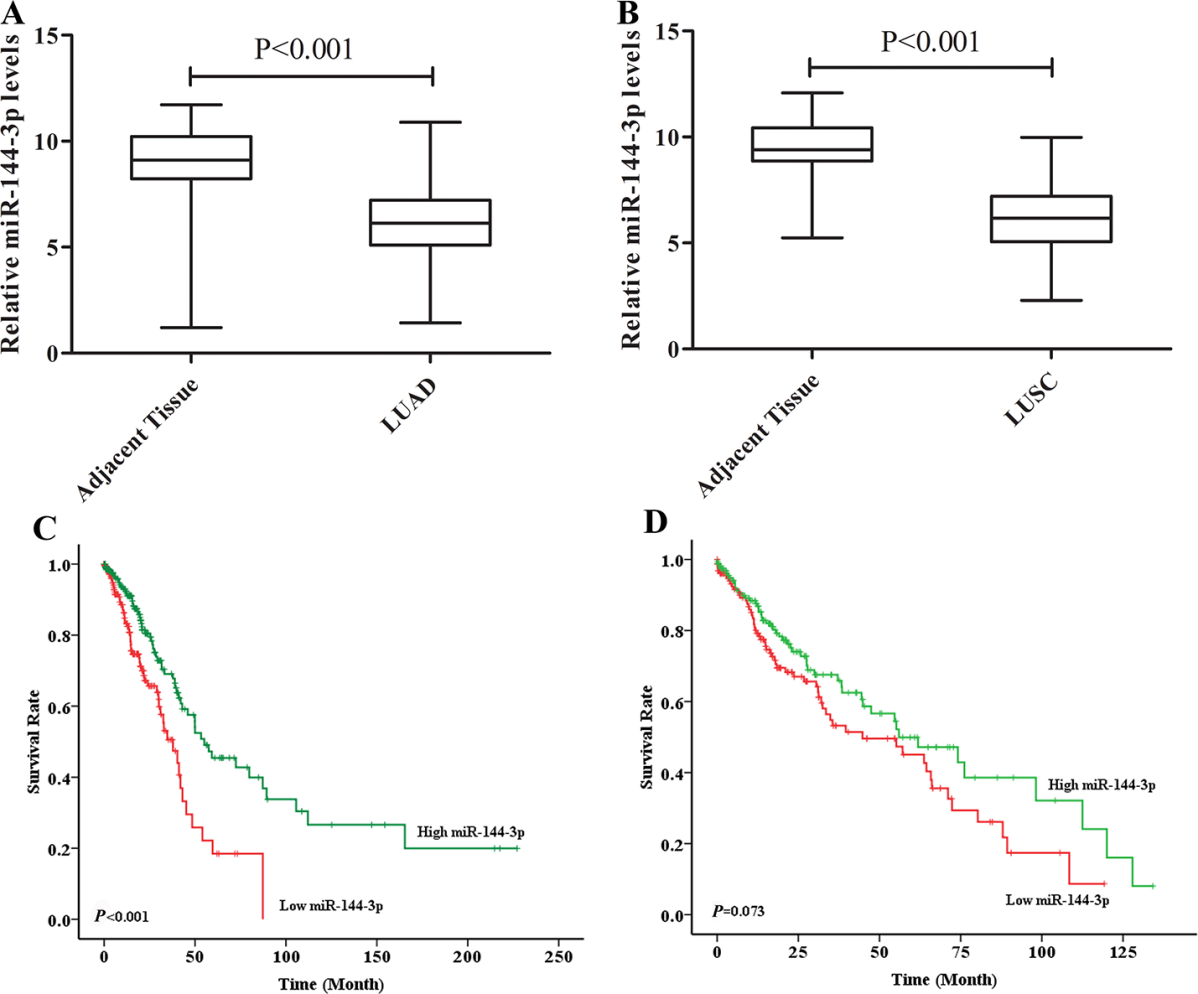
TCGA data validated the correlation of miR-144-3p and prognosis Based on TCGA database, miR-144-3p level in tumor tissue was crucially higher than that in adjacent area in both LUAD (*P <* 0.001) (**A**) and LUSC (*P* < 0.001) (**B**) patients; in LUAD, patients with high miR-144-3p had significantly longer OS than low miR-144-3p group (*P* < 0.001) (**C**), while no distinct prognostic difference was found in LUSC population (*P* = 0.073) (**D**).

In LUAD patients, a negative correlation was detected between IL-1β and miR-144-3p (r = −0.540, *P* < 0.001, Figure 4A), and the high miR-144-3p group had better prognosis than low miR-144-3p group (*P* = 0.003, Figure 4C). In contrast, miR-144-3p was neither correlated with IL-1β (r = −0.159, *P* = 0.250, Figure 4B) nor prognosis (*P* = 0.633, Figure 4D) in LUSC patients. TCGA data displayed that miR-144-3p was significantly higher in both tumor tissues compared with adjacent area in both LUAD (Figure 5A) and LUSC (Figure 5B) populations, but its expression was only associated with prognosis in LUAD patients (Figure 5C and 5D). By χ^2^ test, we confirmed that miR-144-3p was correlated with IL-1β only in LUAD patients (Table 2).

**Table 2:** Correlation between miR-144-3p and clinical features in LUAD and LUSC patients

	LUAD				LUSC			
Characteristics	All patients	miR-144-3p Low (< median^a^)	miR-144-3p High (≥ median^a^)	*p*	All patients	miR-144-3p Low (< median^b^)	miR-144-3p High (≥ median^b^)	*p*
***n***	129 (100%)	64 (49.61%)	65 (50.39%)		54 (100%)	27 (50.00%)	27 (50.00%)	
**Age (years)**				0.982				0.817
** Median (range)**	65 (48–85)	65 (48–85)	65 (49–84)		64 (50–85)	64 (51–85)	64 (50–83)	
**Gender**				0.736				0.362
** Male**	93 (72.09%)	47 (36.43%)	46 (35.66%)		39 (72.22%)	21 (38.89%)	18 (33.33%)	
** Female**	36 (27.91%)	17 (17.18%)	19 (14.73%)		15 (27.78%)	6 (11.11%)	9 (16.67%)	
**Smoke**				0.425				1.000
** Yes**	68 (52.71%)	36 (27.91%)	32 (24.80%)		36 (66.67%)	18 (33.33%)	18 (33.33%)	
** No**	61 (47.29%)	28 (21.71%)	33 (25.58%)		18 (33.33%)	9 (16.67%)	9 (16.67%)	
**Pathological stage^c^**				0.726				0.141
** IA**	17 (13.18%)	10 (7.75%)	7 (5.43%)		11 (20.37%)	3 (5.56%)	8 (14.81%)	
** IB**	34 (26.35%)	14 (10.85%)	20 (15.50%)		10 (18.52%)	6 (11.11%)	4 (7.41%)	
** IIA**	23 (17.83%)	11 (8.53%)	12 (9.30%)		11 (20.37%)	8 (14.81%)	3 (5.56%)	
** IIB**	32 (24.81%)	17 (13.18%)	15 (11.63%)		13 (24.07%)	7 (12.96%)	6 (11.11%)	
** IIIA**	11 (8.53%)	7 (5.43%)	4 (3.10%)		8 (14.81%)	2 (3.70%)	6 (11.11%)	
** IIIB**	12 (9.30%)	5 (3.88%)	7 (5.43%)		1 (1.85%)	1 (1.85%)	0 (0.00%)	
**IL-1β Level**				< 0.001				0.057
** Low**	64 (49.61%)	11 (8.52%)	53 (41.09%)		27 (50.00%)	10 (18.52%)	17 (31.48%)	
** High**	65 (50.39%)	53 (41.09%)	12 (9.30%)		27 (50.00%)	17 (31.48%)	10 (18.52%)	
**Target therapy**				0.660				NT^d^
** Yes**	64 (49.61%)	33 (25.58%)	31 (24.03%)		NT^d^	NT^d^	NT^d^	
** No**	65 (50.39%)	31 (24.03%)	34 (26.36%)		NT^d^	NT^d^	NT^d^	

Mediana^a^ = 8.1731 Median^b^ = 5.8994 NT^d^ = Not Tested

Pathological stage^C^ was based on NCCN Guidelines Version 3.2011 NSCLC.

NCCN, National Comprehensive Cancer Network; NSCLC, non-small-cell lung cancer.

In LUAD patients, univariate analysis showed that age, clinical stage, target therapy, IL-1β, TNF-α and miR-144-3p were significant prognostic factors, while gender and smoking status showed no statistical differences (Table 2). As an association was determined between IL-1β and miR-144-3p, two separate multivariate models (IL-1β and miR-144-3p) were run to avoid problems of multicollinearity. Multivariate analyses demonstrated that clinical stage, IL-1β and miR-144-3p were independent risk factors for OS (Table 3). In LUSC patients, no independent risk factor was verified (Table 4).

**Table 3:** Univariate and multi-variate analysis of the clinicopathological and molecular features for OS in LUAD

Clinicopathological parameters	Comparison reference	Univariate analysis		Multi-variate analysis	
		Hazard ratio (95%CI)	*P* value	Hazard ratio (95%CI)	*P* value
Age	< 65	Ref		Ref	
	≥ 65	1.906 (1.079–3.366)	0.026	1.566 (0.838–2.926)^a^	0.159^a^
Gender	Male	Ref			
	Female	1.176 (0.640–2.160)	0.602		
Smoke	No	Ref			
	Yes	1.577 (0.891–2.790)	0.118		
Clinical Stage	IA	Ref	< 0.001	Ref	< 0.001^a^
	IB	1.058 (0.177–6.338)	0.951	0.907 (0.150–5.499)^a^	0.916^a^
	IIA	4.515 (0.934–21.817)	0.061	4.513 (0.919–22.164)^a^	0.064^a^
	IIB	6.102 (1.397–26.660)	0.016	4.278 (0.966–18.945)^a^	0.056^a^
	IIIA	19.666 (4.234–91.348)	< 0.001	13.101 (2.796–61.386)^a^	0.001^a^
	IIIB	10.963 (2.415–49.760)	0.002	6.050 (1.307–27.996)^a^	0.021^a^
Target therapy	Yes	Ref		Ref	
	No	2.000 (1.129–3.542)	0.018	1.697 (0.934–3.083)^a^	0.083^a^
IL-1β level	Low	Ref		Ref	
	High	3.818 (1.986–7.339)	< 0.001	3.166 (1.592–6.297)^a^	0.001^a^
TNF-α level	Low	Ref		Ref	
	High	1.954 (1.101–3.466)	0.022	1.332 (0.730–2.431)^a^	0.350^a^
miR-144-3p level	Low	Ref		Ref	
	High	0.427 (0.238–0.765)	0.004	0.365 (0.196–0.681)^b^	0.002^b^
Age	< 65	Ref			
	≥ 65	0.649 (0.280–1.503)	0.307		
Gender	Male	Ref			
	Female	0.913 (0.375–2.224)	0.842		
Smoke	No	Ref			
	Yes	0.909 (0.384–2.153)	0.828		
Clinical Stage	IA	Ref	0.039	Ref	0.221
	IB	2.830 (0.255–31.346)	0.397	3.088 (0.275–34.686)	0.361
	IIA	5.361 (0.626–45.921)	0.125	4.805 (0.550–41.949)	0.156
	IIB	9.739 (1.186–79.956)	0.034	8.430 (0.995–71.386)	0.050
	IIIA	17.498 (2.100–145.829)	0.008	13.287 (1.442–122.460)	0.022
	IIIB	19.248 (1.157–320.344)	0.039	14.606 (0.812–262.349)	0.069
IL-1β level	Low	Ref		Ref	
	High	3.106 (1.218–7.917)	0.018	1.531 (0.503–4.657)	0.453
TNF-α level	Low	Ref			
	High	0.810 (0.357–1.839)	0.615		
miR-144-3p level	Low	Ref			
	High	1.216 (0.533–2.777)	0.642		

^a^Multivatiate model including Age, Clinical Stage, Target therapy, IL-1β & TNF-α.

^b^Multivatiate model including Age, Clinical Stage, Target therapy, miR-144-3p & TNF-α.

**Table 4:** Univariate and multi-variate analysis of the clinicopathological and molecular features for OS in LUSC

Clinicopathological parameters	Comparison reference	Univariate analysis		Multi-variate analysis	
		Hazard ratio (95%CI)	*P* value	Hazard ratio (95%CI)	*P* value
Age	< 65	Ref			
	≥ 65	0.649 (0.280–1.503)	0.307		
Gender	Male	Ref			
	Female	0.913 (0.375–2.224)	0.842		
Smoke	No	Ref			
	Yes	0.909 (0.384–2.153)	0.828		
Clinical Stage	IA	Ref	0.039	Ref	0.221
	IB	2.830 (0.255–31.346)	0.397	3.088 (0.275–34.686)	0.361
	IIA	5.361 (0.626–45.921)	0.125	4.805 (0.550–41.949)	0.156
	IIB	9.739 (1.186–79.956)	0.034	8.430 (0.995–71.386)	0.050
	IIIA	17.498 (2.100–145.829)	0.008	13.287 (1.442–122.460)	0.022
	IIIB	19.248 (1.157–320.344)	0.039	14.606 (0.812–262.349)	0.069
IL-1β level	Low	Ref		Ref	
	High	3.106 (1.218–7.917)	0.018	1.531 (0.503–4.657)	0.453
TNF-α level	Low	Ref			
	High	0.810 (0.357–1.839)	0.615		
miR-144-3p level	Low	Ref			
	High	1.216 (0.533–2.777)	0.642		

### The regulatory effect of IL-1β and miR-144-3p on LUAD cell proliferation

**Figure 6:**
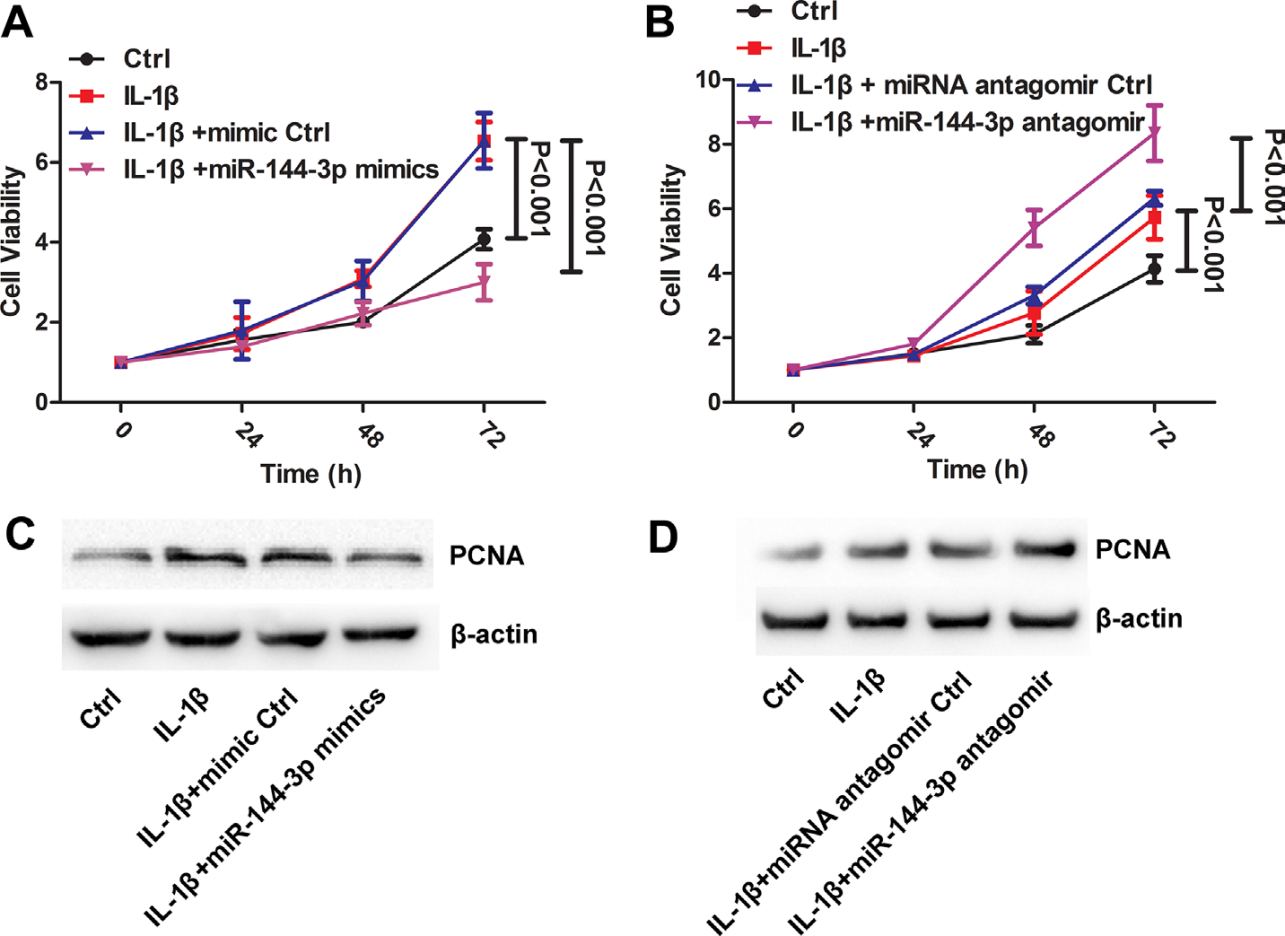
L IL-1β and miR-144-3p were involved in regulation of cell proliferation In A549, IL-1β exposure and miR-144-3p mimics enhanced and suppressed cell proliferation, respectively (*P* < 0.001) (**A**). The combination of IL-1β exposure and miR-144-3p silencing promoted proliferation more significantly than mono-treatment (*P* < 0.0001) (**B**). Western-blot validated MTT result that PCNA protein level was increased after IL-1β exposure (**C** and **D**), while it was increased and decreased after miR-144-3p mimics and antagomir transfection, respectively.

In A549 cells, IL-1β exposure and miR-144-3p mimics enhanced and suppressed cell proliferation, respectively (*P* < 0.001, Figure 6A). The combination of IL-1β exposure and miR-144-3p silencing promoted proliferation more significantly than mono-treatment (*P* < 0.0001, Figure 6B). Western-blot validated MTT result that PCNA protein level was increased after IL-1β exposure (Figure 6C and 6D), while it was increased and decreased after miR-144-3p mimics and antagomir transfection, respectively.

## DISCUSSION

In the present study, we found that the IL-1β level was significantly higher in LUAD and LUSC patients. In LUAD patients, serum IL-1β level was correlated with miR-144-3p, both of which were associated with prognosis. Moreover, IL-1β was involved in regulation of miR-144-3p at the transcriptional level; IL-1β and miR-144-3p over-expression both could promote the cell proliferation.

Cytokines play key roles in initiation and development of cancer [19, 20]. IL-1β has been approved to be an essential factor in the inflammation initiation [9]. Gui et al. demonstrated that IL-1β can trigger HIF-1 through NF-κB/COX-2 signaling pathway, suggesting that HIF-1 is a bridge linking inflammation and tumor and IL-1β possesses a cancer promoting property [21]. IL-1β-induced COX-2 would mediate the evolution from inflammation to cancer through HIF-1, and such a process could be inhibited or suppressed by nonsteroidal anti-inflammatory drugs (NSAIDs), such as Aspirin and Celecoxib, leading to apoptosis of cancer cells [22]. Nevertheless, a large portion of NSCLC patients can not benefit from NSAIDs but suffer tumor progression quickly in clinic [23]. A few studies have approved that Celecoxib can promote epithelial-mesenchymal transition (EMT) of cancer cells, leading to increased metastatic risk [24, 25]. The conflict results indicated that COX-2/HIF1 is probably not the only cancer promoting pathway in downstream of IL-1β. In general, the existing evidence is not convincing enough to elucidate carcinogenetic mechanism of IL-1β, and new breakthroughs are urgently desired.

Despite under the same catalog of NSCLC, LUAD and LUSC greatly differ in genetics, histology, treatment and prognosis. Recently, Campbell JD et al. identified distinct patterns of somatic genome alterations in LUAD and LUSC. The genetics of the two major types of NSCLC significantly vary, which can be defined as individual diseases in certain level. Although targeted therapies for LUAD and LUSC are largely distinct, immunotherapy may aid in treatment for both types [3]. In the present study, we found that only in LUAD, serum IL-1β level was correlated with miR-144-3p, and both of them were independent risk factors for prognosis. However, no such significant prognostic value of IL-1β or miR-144-3p was obtained in LUSC. Our result was validated by TCGA database. Therefore, we suggested to investigate LUAD and LUSC separately to avoid major confounding factors in the future studies.

In this study, IL-8, IL-10 and TNF-α levels in LUSC were lower than those of healthy donors (*P* < 0.001). However, no prognostic value was observed. Although a number of studies have demonstrated the correlation between cytokine levels and prognosis in NSCLC patients, the population size was limited in the present study. The potential reason and mechanism remain unclear, and further investigation is necessary.

Although great efforts have been made to improve diagnosis and effective treatment, the prognosis of lung cancer remains poor due to lack of fine biomarkers. Accumulating evidence has confirmed that miRNAs are involved and play pivotal role in the occurrence, development and prognosis of tumor [26, 27]. Our results indicated that IL-1β probably promoted cell proliferation via suppression of miR-144-3p. Tumor miRNA is ubiquitous and stable in peripheral blood, and it is convenient to detect by real-time PCR or miRNA sequencing [28–30], making it a potential marker with synergetic diagnostic value. miRNA participates in every step of “inflammation--premalignancy--malignancy” process, including cytokine release and immune response [14]. So far, it remains unclear how miRNA mediates and regulates “inflammation-cancer transduction” in lung cancer. Therefore, such an investigation is important for the patients. Based on our findings, we hypothesized that IL-1β might regulate miR-144-3p transcription and control various biological behaviors, and its expression was associated with prognosis in LUAD patients. However, it still remains undefined how miR-144-3p acts and what the cross-talk between miR-144-3p and COX-2/HIF-1 pathway is.

Taken together, we demonstrated that in LUAD patients, the serum IL-1β level was correlated with miR-144-3p, and it could affect miR-144-3p at the transcriptional level. Moreover, both of them were associated with prognosis and independent risk factors for LUAD patients. In addition, IL-1β might promote cell proliferation via suppression of miR-144-3p. As the limit on the sample size, more elaborate studies are necessary for further exploration of the link between IL-1β and miR-144-3p and their roles in tumorigenesis.

## MATERIALS AND METHODS

### Patients, samples and cell line

The human NSCLC cell line A549 was obtained from American Type Culture Collection (ATCC, Manassas, USA) and cultured according to the guideline.

The study was performed on a total of 183 randomly selected NSCLC patients (129 LUADs and 54 LUSCs). Specimens were collected during biopsies and surgery from the Third Affiliated Hospital of Soochow University (Changzhou, China), and freshly collected samples were immediately fixed in formaldehyde. Each patient participated in the study after informed consent was provided. All specimens were histologically and blindly classified by two professional pathologists according to the national NCCN guidelines for NSCLC. Serum samples were collected from the same 183 patients and 40 extra healthy donors and stored in liquid nitrogen.

### Enzyme linked immunosorbent assay (ELISA)

Serum samples from patients and healthy donors were obtained before biopsy or surgery from the Third Affiliated Hospital of Soochow University (Changzhou, China). The levels of cytokines, including IL-1β, IL-4, IL-6, IL-8, IL-10 and TNF-α, were determined using ELISA assay kits according to manufacturerwere determined using ELISA assay kits according tos instructions (R&D, Shanghai, China).

### miRNA isolation

Total RNA was isolated from 400 μL serum of each patient using the miRNeasy Mini Kit (Qiagen, Shanghai, China) according to the manufacturer's instructions. Purified total RNA was used for further real-time PCR and miRNA array test.

### miRNA array

Serum miRNA profile was generated using RiboArray TM miDETECT TM Human Array 1 × 12K (Ribobio, Guangzhou, China) according to the manufacturer's instructions.

### Quantitative reverse transcription PCR (qRT-PCR)

The level of miR-144-3p was quantified by qRT-PCR using Bulge-Loop™ miRNA qRT-PCR Primer Set (Ribobio, Guangzhou, China), and results were analyzed and expressed by ^ΔΔ^CT method.

### The cancer genome atlas (TCGA) database

LUAD and LUSC samples with miR-144-3p expression and clinical information were obtained from TCGA database, yielding a total of 452 LUADs and 343 LUSCs patients. The K-means cluster method (K = 2) was carried out to cluster the patients into two groups based on their miR-144-3p expression. The log-rank test and Kaplan-Meier curve were performed to evaluate the statistical significance and prognosis of the two groups.

### Cell culture, miR-144-3p mimics and antagomir transfection

LUAD cell line A549 was employed for *in vitro* experiments. For treatment, cells were treated with 10 ng/mL IL-1β (PrimeGene, Shanghai, China). Mimics and antagomir were purchased from Ribobio Co., Ltd. (Guangzhou, China) in order to induce and suppress the miR-144-3p expression, respectively.

### MTT assay

Cell proliferation was determined using Vybrant MTT Cell Proliferation Assay Kit (Invitrogen, Shanghai, China). Cells were collected at 0, 24, 48 and 72 h after IL-1β exposure and miR-144-3p mimics/antagomir transfection. Absorbance at a wavelength of 570 nm was determined on a spectrometer. The experiment was performed in triplicates.

### Protein extraction and Western-blot analysis

Total proteins were extracted using CHAPS lysis buffer. Proteins were separated by sodiem dodecyl sulfate polyacrylamide gel electrophoresis and transferred onto polyvinylidene fluoride (PVDF) membrane. Blots were blocked in 4% dry milk at room temperature for 1 h, and immuno-stained with primary antibodies (anti-PCNA, 1:2,000; Cell signaling Technology, Shanghai, China and β-actin 1:10,000; Dako, Glostrup, Denmark) at 4°C overnight. The results were visualized via a chemiluminescent detection system (Pierce ECL Substrate Western-blot detection system; Thermo, Rockford, IL) and exposed in Molecular Imager ChemiDoc XRS System (Bio-Rad, Hercules, CA).

### Statistical analysis

Statistical analysis was performed using GraphPad Prism (version 5.01; GraphPad Software, Inc., La Jolla, USA) and SPSS (version 17, SPSS Inc., Chicago, USA) statistical softwares. The Student's *t* test and paired t test were used to analyze significance between independent groups and paired materials, respectively. The correlation test was used to analyze the correlation between IL-1β and miR-144-3p. The χ^2^ test was used to test the significance of observed differences in proportions except when the cell size was less than 5 (Fisher's exact tests). Survival curves were made using the Kaplan-Meier method and compared using the log-rank test. All variables that achieved a significance at *P* ≤ 0.05 in univariate analyses were enrolled in multivariate Cox's proportional hazard model. *P* ≤ 0.05 was considered statistically significant unless otherwise specified.
